# Inhibitory Effects of 3-(4-Hydroxy-3-methoxyphenyl) Propionic Acid on Amyloid β-Peptide Aggregation In Vitro

**DOI:** 10.3390/biomedicines13071649

**Published:** 2025-07-06

**Authors:** Makoto Mori, Hiroto Nakano, Sadao Hikishima, Jota Minamikawa, Daiki Muramatsu, Yasuhiro Sakashita, Tokuhei Ikeda, Moeko Noguchi-Shinohara, Kenjiro Ono

**Affiliations:** Department of Neurology, Kanazawa University Graduate School of Medical Sciences, Kanazawa 920-8640, Japan; m0229maa@med.kanazawa-u.ac.jp (M.M.); neuro-nakano@med.kanazawa-u.ac.jp (H.N.); s-hikishima@med.kanazawa-u.ac.jp (S.H.); jota373@med.kanazawa-u.ac.jp (J.M.); d.mura@med.kanazawa-u.ac.jp (D.M.); skst@med.kanazawa-u.ac.jp (Y.S.); tokuhei@med.kanazawa-u.ac.jp (T.I.); m-nohara@med.kanazawa-u.ac.jp (M.N.-S.)

**Keywords:** 3-(4-hydroxy-3-methoxyphenyl) propionic acid, amyloid β-protein, aggregation, nucleation, mature fibril Aβ seeds

## Abstract

**Objectives**: The compound 3-(4-Hydroxy-3-methoxyphenyl) propionic acid (HMPA) is a terminal metabolite derived from polyphenol compounds. It has been studied for its potential to support brain health indirectly through its anti-oxidant effects and ability to enhance the gut environment; however, its role in dementia pathogenesis is unclear. Therefore, the aim of this study was to evaluate how HMPA inhibits Aβ42 aggregation in vitro. **Methods**: We examined the inhibitory effects of HMPA on amyloid-β protein (Aβ) aggregation using a thioflavin T (ThT) assay and electron microscopy (EM). **Results**: ThT assays demonstrated that HMPA inhibited both the nucleation and elongation phases of Aβ aggregation. Additionally, EM of low-molecular-weight (LMW) Aβ42 in the presence of HMPA demonstrated shorter fibrils compared to those formed without HMPA. The EC_50_ of HMPA in LMW Aβ42 was 5–6 mM. **Conclusions**: These findings indicate that, similar to several polyphenol compounds such as myricetin and rosmarinic acid, HMPA may inhibit Aβ pathogenesis, although it requires a fairly high concentration in vitro. These findings suggest the potential of HMPA as a lead compound for modulating Aβ-related neurodegeneration.

## 1. Introduction

Dementia is a comprehensive disorder characterized by a reduction in acquired cognitive function [1]. The population of patients with dementia is estimated to exceed 152.8 million by 2050 [2]. Most dementia cases are caused by neurodegenerative disorders, such as Alzheimer’s disease (AD), dementia with Lewy bodies, and frontotemporal lobar degeneration [1]. AD is the most common cause of dementia, affecting a growing number of people worldwide [1], with approximately 5–6% of individuals aged over 65 years diagnosed with AD [1]. Extracellular accumulation of amyloid-β protein (Aβ), known as senile plaques, and intracellular accumulation of phosphorylated tau—neurofibrillary tangles— are the primary pathological features of AD [3,4]. Aβ—the primary pathogenic protein in AD—has two major isoforms: Aβ40 and Aβ42 [5,6]. In 1992, Hardy and Higgins [7] proposed the amyloid cascade hypothesis, indicating that extracellular accumulation of Aβ42 induces synaptic dysfunction and neuronal cytotoxicity through abnormally phosphorylated tau, resulting in neuronal cell death and cognitive impairment. The reduction in senile plaques in the brains of patients with AD did not enhance the cognitive dysfunction, indicating that mature fibrils (MFs) may not be the most neurotoxic species in Aβ aggregation [8]. Soluble oligomers may be more likely to initiate synaptic and neuronal dysfunction rather than MFs [9]. Currently, oligomeric species are regarded as the most neurotoxic species in the amyloid cascade, and Aβ protofibrils—which constitute a subtype of high-molecular-weight oligomers—have been recognized as therapeutic targets for patients with AD [10]. The development of anti-Aβ monoclonal antibodies represents the latest advancement in AD treatment. Lecanemab, which targets Aβ protofibrils, has been developed and is being used globally. Different types of Aβ protofibrils have been identified based on species morphology, including globular, annular, and curvilinear forms. Notably, globular-shaped Aβ oligomer facilitated fibril formation in low-molecular-weight (LMW) Aβ by accelerating its fibril elongation [11]. Despite advances in the development of novel drugs for patients with AD, a definitive cure for AD pathogenesis remains elusive. Therefore, it is crucial to establish innovative methods for treating patients with dementia.

**Figure 1:**
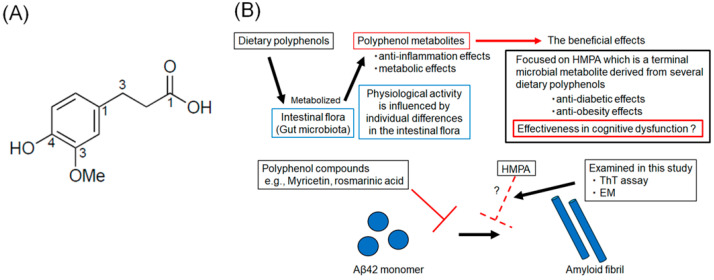
(**A**) The chemical structure of 3-(4-hydroxy-3-methoxyphenyl) propionic acid. (**B**) Schematic illustration of the rationale of this study.

Several polyphenol compounds, extracted from dietary products, such as wine, green tea, and fruits, have demonstrated the potential to prevent the progression of AD pathology [12,13,14,15]. Several health benefits of polyphenol compounds have been reported, including anti-oxidant, anti-tumor, anti-inflammatory, and cardioprotective effects [16,17]. The neuroprotective effects of polyphenol compounds, such as myricetin (Myr) and rosmarinic acid (RA), have been previously reported [18,19]. In in vitro studies, Myr or RA were found to inhibit Aβ-oligomerization and reduce cellular toxicity and synaptic dysfunction [17]. Recently, polyphenolic compounds that inhibit aggregation not only of Aβ but also of tau have been reported, and the search for new polyphenolic compounds that can act AD therapeutics remains an active area of research [15,20]. Although essential for maintaining body health, most polyphenol compounds cannot be absorbed in their native form and should be hydrolyzed by intestinal flora [21]. The composition of the intestinal flora varies among individuals, which influences polyphenol metabolism and, consequently, the biological activity of polyphenols [22]. Polyphenol metabolites exhibit anti-inflammatory and metabolic effects and are recognized as postbiotics that confer health benefits [23,24]. Therefore, it is crucial to assess the inhibitory effects of their metabolic end products. The compound 3-(4-hydroxy-3-methoxyphenyl) propionic acid (HMPA) (Figure 1A) is a terminal microbial metabolite derived from curcumin, γ-oryzanol, and hesperetin [25,26]. Based on this, we hypothesized that HMPA, a final metabolite of certain polyphenols, acts as the core component responsible for the physiological activity of polyphenols. Although previous studies have reported its anti-diabetic and anti-obesity effects, its effectiveness in improving cognitive dysfunction remains unclear [27,28]. In this study, we focused on HMPA and assessed the inhibitory effects of HMPA on Aβ42 aggregation using thioflavin T (ThT) assays and electron microscopy (EM) (Figure 1B).

Many dietary polyphenols are metabolized by the intestinal flora, but there is individual variation in the effects of the intestinal flora, which influences the metabolism and physiological activity of polyphenols. Polyphenol metabolites have several beneficial effects, including anti-inflammatory and metabolic effects. We focused on 3-(4-hydroxy-3-methoxyphenyl) propionic acid (HMPA), a final metabolite of several polyphenols. HMPA is known to have anti-diabetic and anti-obesity effects, but its efficacy for cognitive dysfunction remains unclear. This study investigates whether HMPA can inhibit the aggregation of amyloid-β (Aβ42) in vitro.

## 2. Materials and Methods

### 2.1. Reagents

We used 3-(4-Hydroxy-3-methoxyphenyl) propionic acid (purity ≥ 98%; Tokyo Chemical Industry Co., Ltd. [TCI], Tokyo, Japan) in this study, which has a molecular weight of 196.2 g/mol.

### 2.2. Preparation of Aβ42

Synthetic Aβ42 peptide lyophilizates (trifluoroacetate form, purity ≥ 95.0% assessed using high-performance liquid chromatography) were purchased from the Peptide Institute, Inc. (Osaka, Japan, cat. no. 4349-v). We prepared 200 μL of the Aβ42 peptide in dimethyl sulfoxide at a concentration of 2 mg/mL. After sonication for 1 min using a bath sonicator (Branson Ultrasonics, Danbury, CT, USA), the Aβ42 peptide solution was vortexed at 1500 rpm, transferred to a low-binding tube, and centrifuged (10 min, 16,000× *g*, 15 °C). The supernatant was fractionated using a Superdex 75 HR column in 10 mM phosphate buffer (pH 7.4) at a flow rate of 0.5 mL/min. We collected monomeric species (LMW Aβ42). A 10 μL aliquot was used for amino acid analysis to determine the peptide concentration. The LMW Aβ42 sample was stored at −80 °C.

### 2.3. Preparation of Mature Fibril Aβ42 Seeds

The mature fibril (MF) Aβ42 seeds were prepared as described previously [11]. The Aβ42 peptide lyophilizates were initially dissolved in 0.02% ammonia to yield a 250 μM solution. This was subsequently diluted tenfold with 10 mM phosphate-buffer solution (pH 7.4), resulting in a final Aβ42 concentration of 25 μM. The mixture underwent sonication for 1 min using a Vibra-Cel ultrasonic disruptor (SONICS & MATERIALS, Inc., Newtown, TX, USA), followed by centrifugation at 16,000× *g* for 10 min at 4 °C. The supernatant containing soluble Aβ42 was collected and incubated at 37 °C for 48 h to allow aggregation. Afterward, the samples were sonicated for 30 s on ice and stored at −80 °C as MF seed stock.

### 2.4. ThT Fluorescence Assay

A 300 μL aliquot of mixed Aβ samples was prepared, containing 5 µM LMW Aβ42 with different concentrations of HMPA (5 µM, 1.25 mM, 2.5 mM, 5 mM, and 50 mM), 100 mM NaCl, and 5 µM ThT. Fluorescence was determined thrice at 10 min intervals from 0 to 24 h using a Varioskan lux instrument (Thermo Fisher Scientific, Waltham, MA, USA). Fluorescence measurements were performed using excitation and emission wavelengths of 440 nm and 485 nm, respectively. The final fluorescence intensity for each sample was obtained by averaging three replicate measurements and subtracting the background signal from the ThT-only control.

To assess the kinetics under secondary nucleation-dominant conditions [29], a 300 μL aliquot of mixed Aβ was prepared, containing 5 µM LMW Aβ42, 0.1 µM MF seeds (2% by volume) with different concentrations of HMPA (5 µM, 1.25 mM, 2.5 mM, 5 mM, and 50 mM), 100 mM NaCl, and 5 µM ThT. Additionally, to assess the effects of HMPA on seeding activity-dominant conditions, a similar mixture was prepared with 0.5 µM MF seeds.

To assess the aggregation potency of Aβ42, we used the 50% seeding dose (SD_50_), defined as the time at 50% of the peak value of the targeted proteins during kinetic analysis [11,30]. We calculated the SD_50_ for each sample using Igor Pro version 8 software (HULINKS Inc., Tokyo, Japan). We plotted three data points for each sample in a bar graph with standard deviations.

### 2.5. Assessment of Half Maximal Effective Concentrations (EC_50_) of HMPA

EC_50_ refers to the concentration of a drug or chemical substance required to achieve 50% of its maximum effect [31]; here, it was defined as the HMPA concentration that inhibits fibril formation by 50% [31]; thus, a substance with a lower EC_50_ value is considered to exhibit significant inhibitory effects [32]. EC_50_ values were calculated by fitting the data to a sigmoidal curve using GraphPad Prism software (version 8.0.2, GraphPad Software, Boston, MA, USA).

### 2.6. Assessment of Fibril Formation Using EM

Two types of Aβ samples were prepared: one containing only LMW Aβ42, and another supplemented with 5 mM HMPA. Each sample (100 µL) was incubated at 37 °C for 24 h. Following incubation, 20 µL portions (15 µM in 10 mM phosphate buffer, pH 7.4) were placed onto glow-discharged carbon-coated formvar grids (Okenshoji Co., Ltd., Tokyo, Japan; cat.no. 10-1009) and left at ambient temperature for 20 min. Afterward, the liquid on the grids was carefully replaced with 20 µL of 2.5% (*v*/*v*) glutaraldehyde (Wako Pure Chemical Industries Ltd., Osaka, Japan; cat.no. 073-00536), followed by a 4 min fixation. Grids were subsequently stained using 15 µL of 1% (*v*/*v*) uranyl acetate dihydrate (Wako Pure Chemical Industries Ltd., Osaka, Japan; cat.no. 94260). After removing the solutions, the grids were air-dried and observed for fibrillar structures in each sample using a Hitachi H-7650 transmission electron microscope (Hitachi High-Tech Science, Tokyo, Japan). Representative fibrillar structures were captured, and the morphological features of individual aggregates in EM images were assessed at 15,000× magnification.

### 2.7. Statistical Analysis

To assess the statistical significance of SD_50_ among six different samples, LMW Aβ42 without HMPA was compared to samples containing different concentrations of HMPA (5 µM, 1.25 mM, 2.5 mM, 5 mM, and 50 mM) using Dunnett’s test, that was performed using a GraphPad Prism software (version 8.0.2, GraphPad Software, Boston, MA, USA).

## 3. Results

### 3.1. Kinetic and Morphological Analysis of LMW Aβ42 in the Absence of HMPA

**Figure 2:**
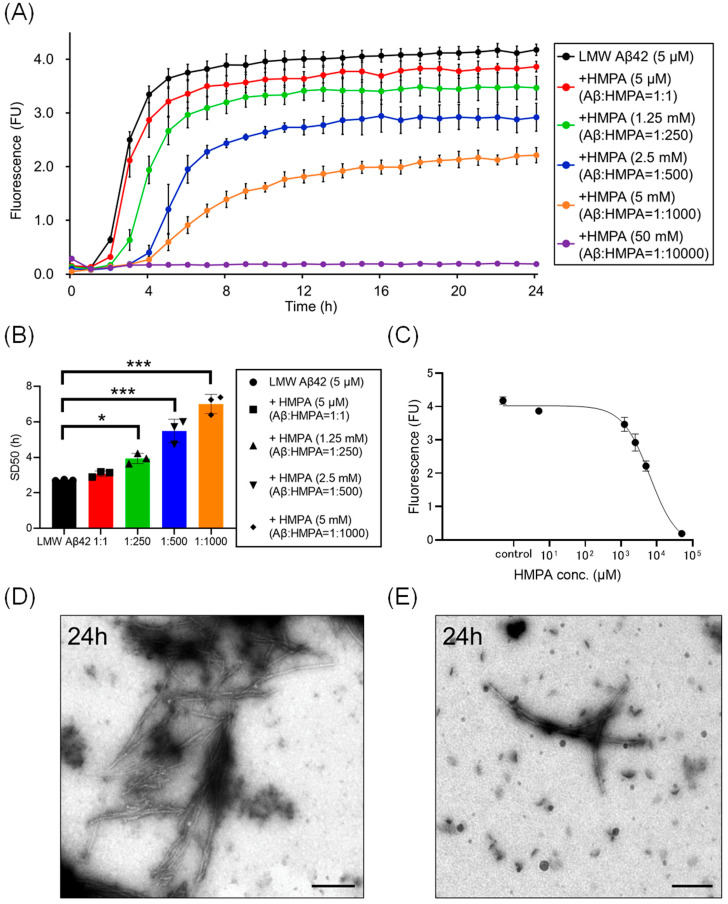
Kinetic analysis of Aβ42 aggregation in the presence of 3-(4-hydroxy-3-methoxyphenyl) propionic acid. Aβ aggregation results in low-molecular-weight (LMW) Aβ42 with various concentrations of 3-(4-hydroxy-3-methoxyphenyl) propionic acid (HMPA) (5 µM, 1.25 mM, 2.5 mM, 5 mM, 50 mM) are displayed (**A**). Each 50% seeding dose (SD_50_) of the mixed sample containing LMW Aβ42 with HMPA is shown in the bar graph (**B**). The EC_50_ of HMPA in LMW Aβ42 is 6370 µM (**C**). Many non-branched fibrils were found with LMW Aβ42 (**D**). By contrast, shorter length-objects were observed in LMW Aβ42 with HMPA (**E**). Scale bar = 250 nm. Asterisks indicate statistical significance of the difference between the two data sets (* *p* < 0.05, *** *p* < 0.001) (one-way analysis of variance [ANOVA] with Dunnett’s test). The black bars in the electron microscopy images indicate a scale of 250 nm.

We analyzed temporal alterations in ThT fluorescence of LMW Aβ42 or without HMPA. The ThT fluorescence of LMW Aβ42 without HMPA demonstrated a sigmoidal curve (Figure 2A). Upon adding different concentrations of HMPA (5 µM, 1.25 mM, 2.5 mM, 5 mM, and 50 mM) to LMW Aβ42, the sample with 50 mM HMPA demonstrated no increase in ThT fluorescence, whereas the other samples demonstrated sigmoidal curves (Figure 2A). We observed HMPA concentration-dependent reductions in the plateau values of ThT fluorescence (Figure 2A). The SD_50_ values of LMW Aβ42 without HMPA were significantly shorter than those of LMW Aβ42 with HMPA (1.25 mM, 2.5 mM, and 5 mM) (*p* < 0.015) (Figure 2B). We could not analyze the SD_50_ of LMW Aβ42 in the presence of HMPA (50 mM) as the sigmoidal curve fittings had failed. Based on the EC_50_ results, the HMPA concentration at 50% of the peak value of the sigmoidal curve in the kinetic assays was 6370 µM (Figure 2C). EM images revealed numerous non-branched fibrils in LMW Aβ42 (Figure 2D). By contrast, shorter length-objects were observed for LMW Aβ42 with HMPA (Figure 2E). The number of fibril structures was reduced in LMW Aβ42 with HMPA (Figure 2E).

### 3.2. Temporal Alterations in the ThT Fluorescence of LMW Aβ42 and 2% MF Seeds in the Presence of HMPA

**Figure 3:**
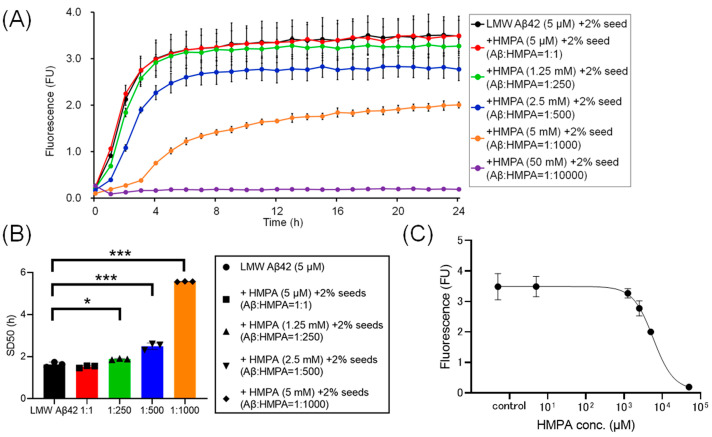
Temporal changes in Aβ42 aggregation in the presence of 3-(4-hydroxy-3-methoxyphenyl) propionic acid and 2% mature fibril Aβ42 seeds. The results of the Aβ aggregation in low-molecular-weight (LMW) Aβ42 in the presence of 2% mature fibril Aβ42 (MF) seeds with various concentrations of 3-(4-hydroxy-3-methoxyphenyl) propionic acid (HMPA) (5 µM, 1.25 mM, 2.5 mM, 5 mM, 50 mM) are shown (**A**). Each 50% seeding dose (SD_50_) of the mixed sample containing LMW Aβ42 with HMPA is shown in the bar graphs (**B**). The EC_50_ of HMPA in LMW Aβ42 is 5779 µM (**C**). Asterisks indicate the statistical significance of the difference between the two data sets (* *p* < 0.05, *** *p* < 0.001).

To assess the kinetics under different sample conditions with 2% MF seeds, we analyzed the ThT kinetics of LMW Aβ42 and 2% MF seeds with or without HMPA. The mixed sample containing LMW Aβ42 with 2% MF seeds and 50 mM HMPA demonstrated no increase in ThT fluorescence, whereas the other mixed samples demonstrated sigmoidal curves in the ThT assay (Figure 3A). The sigmoidal curves among LMW Aβ42 and 2% MF seeds with or without HMPA (5 µM and 1.25 mM) demonstrated similar kinetic curves (Figure 3A). By contrast, the plateau values of ThT fluorescence in LMW Aβ42 and 2% MF seeds with HMPA (2.5 and 5 mM) were reduced compared to those of LMW Aβ42 and 2% MF seeds without HMPA (Figure 3A). The SD_50_ values of LMW Aβ42 with 2% MF seeds in the absence of HMPA were significantly lower than that of LMW Aβ42 with 2% MF seeds when HMPA was present (1.25 mM, 2.5 mM, and 5 mM) (*p* < 0.021) (Figure 3B). We could not analyze the SD_50_ of LMW Aβ42 with 2% MF seeds in the presence of HMPA (50 mM), as the sigmoidal curve fittings had failed. Based on the EC_50_ results, the HMPA concentration at 50% of the peak value of the sigmoidal curve in the kinetic assays was 5779 µM (Figure 3C).

### 3.3. Dynamic Alterations in the ThT Fluorescence of LMW Aβ42 with 10% MF Seeds in the Presence of HMPA

**Figure 4:**
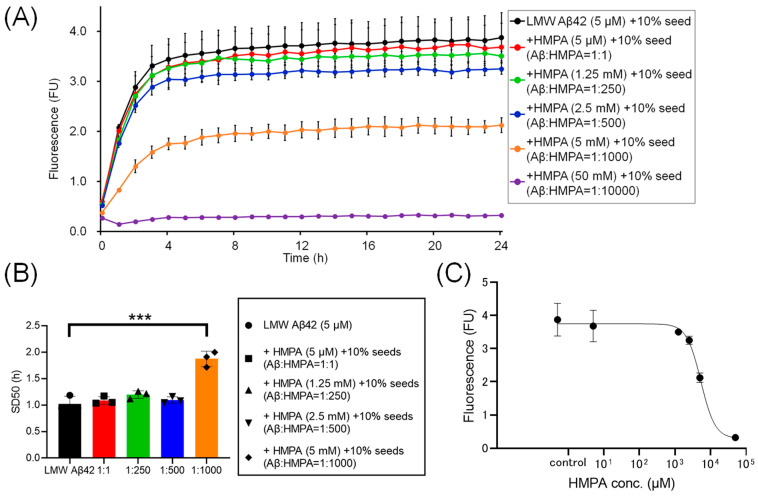
The temporal changes in Aβ42 aggregation in the presence of 3-(4-hydroxy-3-methoxyphenyl) propionic acid and 10% mature fibril Aβ42 seeds. Aβ aggregation results in low-molecular-weight (LMW) Aβ42 with 10% mature fibril Aβ42 (MF) seeds with various concentrations of 3-(4-hydroxy-3-methoxyphenyl) propionic acid (HMPA) (5 µM, 1.25 mM, 2.5 mM, 5 mM, 50 mM) are shown (**A**). Each 50% seeding dose (SD_50_) of the mixed sample containing LMW Aβ42 with HMPA is shown in the bar graph (**B**). The EC_50_ of HMPA in LMW Aβ42 is 5328 µM (**C**). Asterisks indicate the statistical significance of the difference between the two data sets (*** *p* < 0.001).

To assess the kinetics under another sample condition containing 10% MF seeds, we analyzed the ThT kinetics of LMW Aβ42 and 10% MF seeds with or without HMPA. The mixed sample containing LMW Aβ42 with 10% MF seeds and 50 mM HMPA demonstrated no increase in ThT fluorescence, whereas the other mixed samples demonstrated sigmoidal curves (Figure 4A). The sigmoidal curves among LMW Aβ42 and10% MF seeds with or without HMPA (5 µM and 1.25 mM) demonstrated similar kinetic curves (Figure 4A). In contrast, the plateau values of ThT fluorescence in LMW Aβ42 and 10% MF seeds when HMPA (2.5 and 5 mM) was included were reduced compared to those of LMW Aβ42 and 10% MF seeds without HMPA (Figure 4A). The SD_50_ values of LMW Aβ42 with 10% MF seeds without HMPA were significantly lower than those of LMW Aβ42 with 10% MF seeds and HMPA (5 mM) (*p* < 0.0001) (Figure 4B). Additionally, we were unable to analyze the SD_50_ in LMW Aβ42 with 10% MF seeds and HMPA (50 mM) because the sigmoidal curve fittings had failed. Thus, based on the EC_50_ results, the HMPA concentration at 50% of the peak value of the sigmoidal curve in the kinetic assays was 5328 µM (Figure 4C).

## 4. Discussion

Here, the ThT assay results indicated that HMPA exerted a concentration-dependent decrease in ThT fluorescence in LMW Aβ42 when MF seeds were not included (Figure 2A). Similar results were observed in the mixed Aβ samples containing different concentrations of MF seeds (2% and 10%) (Figure 3A and Figure 4A). SD_50_ values for LMW Aβ42 in the presence of higher HMPA concentrations were higher than those in the absence of HMPA (Figure 2B, Figure 3B and Figure 4B). The EC_50_ values were higher than those of Aβ concentrations in the central nervous system [33] (Figure 2C, Figure 3C and Figure 4C).

Polyphenol compounds, such as myr and epigallocatechin-3-gallate, can inhibit Aβ aggregation [34]. However, our study confirmed the inhibitory effects of HMPA under various sample conditions. Incubating LMW Aβ42 in the absence of MF seeds resulted in a primary nucleation-dominant reaction, while there was a secondary nucleation-dominant reaction with co-incubation of LMW Aβ42 and 2% MF seeds [29]. Co-incubation of 10% MF seeds with LMW Aβ42 demonstrates seeding activity-dominant conditions [11,30]. Both the nucleation and fibril elongation phases are major kinetic components of Aβ aggregation [35], which was assessed based on the plateau values and lag time duration in the sigmoid curve [35]. ThT assay results demonstrated that HMPA may inhibit Aβ42 aggregation regardless of the MF seed concentration in the reaction buffers. ThT assays demonstrated longer lag times and lower plateau values in terms of ThT fluorescence in LMW Aβ42 with HMPA than that in LMW Aβ42 without HMPA. These findings indicate that HMPA may interact with both the nucleation and fibril elongation phases, although its precise molecular mechanisms in Aβ aggregation remain unclear.

Most polyphenol compounds exhibit physiological activity after being metabolized in the body. HMPA is a major end product of such metabolism, including that of ferulic acid, curcumin, and chlorogenic acid [36]. HMPA is primarily produced following the ingestion of polyphenol-rich foods, such as whole-wheat flour and coffee [37]. To consider the in vivo effects of polyphenol compounds on both Aβ aggregation and dementia pathogenesis, it is clinically crucial to focus on the final metabolites, which include HMPA, compared to the pre-metabolic compounds studied previously. However, the in vitro assays conducted in this study showed the EC_50_ of HMPA was approximately 5–6 mM, suggesting that relatively high concentrations are required for an effect. This finding is inconsistent with the generally low plasma concentrations resulting from consuming dietary polyphenols, which are typically in the low micromolar range [38,39]. Such concentrations are far lower than the HMPA concentrations that demonstrated efficacy in this study. This suggests that the physiological significance of our findings may be somewhat limited.

In this study, we primarily assessed the ability of HMPA to inhibit Aβ aggregation using a ThT fluorescence assay and transmission EM. We also attempted to investigate the effects of HMPA on Aβ oligomerization and secondary structure in more detail using photo-induced cross-linking of unmodified proteins (PICUP) and circular dichroism (CD) spectroscopy. However, HMPA interfered with the PICUP photochemical crosslinking reaction, and the CD spectra were not reproducible under our experimental conditions. As a result, we were unable to include those findings as definitive data in the present study. Future work should include time-resolved analyses of HMPA’s inhibitory effects on specific Aβ oligomeric species, particularly dimers and trimers, which are known to exhibit higher neurotoxicity. ThT assays using well-defined oligomers, combined with high-resolution structural techniques such as atomic force microscopy, would provide deeper insight into how HMPA modulates the early stages of Aβ aggregation. Although these issues remain, we believe that multi-modal analyses of how terminal metabolite polyphenol compounds such as HMPA affect Aβ aggregation will be crucial in future studies.

## 5. Conclusions

The in vitro analysis in this study demonstrated that HMPA exhibits a concentration-dependent inhibition of the aggregation of LMW Aβ42. The estimated EC_50_ value was approximately 5–6 mM, and delayed fibril formation and reduced overall aggregation were observed under various seeding conditions. These results suggest that HMPA may interfere with the nucleation and fibril elongation phases of Aβ aggregation. Although the effective concentration exceeds physiological levels, this study demonstrates the potential of polyphenol-derived metabolites as lead compounds for anti-amyloid aggregation strategies, promoting the search for novel compounds.

## Data Availability

The data that support the findings of this study are openly available on the OSF site at https://osf.io/tpz7j/ (accessed on 23 June 2025).

## Abbreviations

The following abbreviations are used in this manuscript:

AD	Alzheimer’s disease
Aβ	Amyloid-β protein
LMW	Low molecular weight
Myr	Myricetin
RA	Rosmarinic acid
HMPA	3-(4-hydroxy-3-methoxyphenyl) propionic acid
ThT	Thioflavin T
EM	Electron microscopy
MF	Mature fibril Aβ42
SD_50_	50% seeding dose
EC_50_	Half maximal effective concentrations
